# War‐Related Posttraumatic Stress Symptoms During the First Two Postpartum Years: The Moderating Role of Childhood Adversity

**DOI:** 10.1002/smi.70216

**Published:** 2026-08-23

**Authors:** Miriam Chasson, Ayelet David, Dana Shai, Jessica L. Borelli

**Affiliations:** ^1^ Child Development Program Faculty of Education Bar‐Ilan University Ramat Gan Israel; ^2^ Gonda Multidisciplinary Brain Research Center Bar‐Ilan University Ramat Gan Israel; ^3^ Harris Program for Infants, Toddlers, and Their Families Bar Ilan University Ramat Gan Israel; ^4^ The Relational Lab Academic College Tel‐Aviv‐Yaffo Tel Aviv‐Yafo Israel; ^5^ Department of Psychology University of California Irvine California USA

**Keywords:** adverse childhood experiences, mothers, posttraumatic stress symptoms, stress sensitisation, trauma, war

## Abstract

Early motherhood is a sensitive period marked by increased psychological vulnerability, potentially impacting child developmental trajectories. While childhood adversity and war‐related trauma are known as risk factors for psychopathology, little is known about their combined impact on posttraumatic stress symptoms (PTSS) during the first 2 years of motherhood. Grounded in stress sensitisation, this two‐wave study examined how childhood adverse experiences (ACEs) and childhood war‐related insecurity moderate the prospective association between contemporary cumulative war exposure and maternal PTSS. A sample of 394 Israeli mothers was assessed at two time points during the Israel–Hamas war: T1 (5 months post‐outbreak) and T2 (14 months later). Participants were categorised into a four‐group typology based on developmental history: No adversity, ACEs only, Childhood war‐related insecurity only, and Combined (ACEs + Childhood war‐related insecurity). High symptom stability was observed, yet mothers in the Combined group exhibited the highest clinical risk, with 41.7% meeting the PTSD threshold at T2. Additionally, childhood adversity moderated the link between contemporary cumulative war exposure and PTSS at T2. Contemporary cumulative war exposure was associated with a steeper increase in PTSS primarily among mothers in the Combined group, supporting the stress sensitisation model. Mothers with a dual history of interpersonal adversity and childhood war‐related insecurity appeared particularly sensitised to renewed threats. These results highlight the necessity of trauma‐informed care and screening to support maternal and infant well‐being in conflict zones.

## 1 Introduction

Exposure to collective violence, including war and terrorism, constitutes a high‐intensity external stressor and has been consistently linked to elevated rates of posttraumatic stress symptoms (PTSS) among adults residing in conflict‐affected regions (Amsalem et al. 2025; Lim et al. 2022; Palgi et al. 2026). According to the DSM‐5 (American Psychiatric Association 2013), PTSS is characterised by intrusive re‐experiencing, avoidance of trauma‐related cues, negative alterations in cognition and affect, and heightened arousal following exposure to traumatic events. Early motherhood constitutes a developmental transition marked by increased psychological sensitivity and elevated vulnerability to mental health difficulties (Gelaye et al. 2016; Miller et al. 2024; Oh et al. 2016). During this period, war‐related exposure may be especially challenging, as personal threats, disruption of daily routines, chronic uncertainty, and heightened concerns regarding child safety converge with the demands of caring for an infant (Chasson, Borelli, et al. 2025; Kaitz et al. 2009; Osofsky 1995).

Growing evidence indicates that war‐related stress is associated with disruptions in parenting, with implications for children's adjustment (Eltanamly et al. 2021; Levavi et al. 2024; McElroy et al. 2024; Sim et al. 2018). Within this broader literature, maternal PTSS following war‐related trauma has been linked to caregiving‐related alterations, including reduced emotional availability and disruptions in parent–infant interaction, with potential consequences for infants' biological and socioemotional development (Chasson et al. 2025; Feldman and Vengrober 2011; Qouta et al. 2021; Slone and Mann 2016; Van Ee et al. 2012). Consistent with these findings, baseline data from the present longitudinal cohort demonstrated that maternal war‐related PTSS was associated with impaired mother–infant bonding (Chasson et al. 2025). Accordingly, identifying the factors associated with maternal PTSS in the context of ongoing war has become an important research priority, given its implications for maternal mental health, the mother–infant relationship, and early child development.

One factor that may help explain differences in PTSS levels is childhood adversity. Adverse childhood experiences (ACEs) refer to potentially traumatic experiences that occur before the age of 18, including abuse, neglect, and exposure to household dysfunction (Felitti et al. 1998). A substantial body of research has demonstrated robust, dose–response associations between cumulative exposure to ACEs and adverse mental health outcomes in adulthood, including elevated psychological distress and PTSS (Felitti et al. 1998; Hughes et al. 2017). Theoretical perspectives differ in their predictions of how early adversity may be associated with responses to subsequent stressors. While some perspectives suggest that exposure to manageable adversity may, under certain conditions, promote subsequent adaptation or resilience (Rutter 2012; Seery et al. 2010), stress sensitization and stress amplification perspectives propose that more severe or chronic adversity may increase vulnerability when individuals encounter later stressors (Hammen et al. 2000; McLaughlin et al. 2010; Monroe and Harkness 2005). These differing theoretical perspectives underscore the importance of empirically investigating how childhood adversity is associated with psychological responses to major stressors encountered later in life.

Within the context of early motherhood, a growing body of evidence, including meta‐analytic findings, indicates that women with a history of ACEs are at heightened risk for postpartum psychopathology, including depression, anxiety, and PTSS (Fu et al. 2024; Racine et al. 2021). Beyond increasing vulnerability to psychopathology, ACEs have also been linked to difficulties in emotion regulation, elevated parenting stress, and less optimal parent–infant interactions (Fuchs et al. 2015; Lange et al. 2019; Rowell and Neal‐Barnett 2022). Together, these findings suggest that early motherhood may represent a developmental context in which stress‐related vulnerabilities associated with earlier adverse experiences become particularly salient (Brand et al. 2010; Duguay et al. 2025).

Longitudinal evidence from Israel following the October 7, 2023, attack highlights substantial heterogeneity in posttraumatic symptom trajectories, with individuals exposed to higher levels of childhood adversity and greater war‐related stress being more likely to exhibit chronic or escalating PTSS over time (Levin et al. 2025). To date, however, this line of research has rarely focused on women during early motherhood, despite their heightened psychological sensitivity. Moreover, while cumulative ACEs scores capture overall exposure burden, specific forms of childhood adversity may be particularly relevant in contexts of renewed threat (McLaughlin et al. 2021; Pynoos et al. 1999). Specifically, childhood experiences characterized by chronic fear and a lack of or inadequate protection have been linked to enduring feelings of insecurity and heightened threat sensitivity throughout development (McLaughlin and Lambert 2017; Pynoos et al. 1999).

Recent longitudinal evidence further underscores this, demonstrating that early‐life unpredictability, characterized by frequent environmental and familial changes, not only correlates with higher baseline psychological distress but also predicts a sharper increase in emotion dysregulation and distress in response to the October 7 attacks (Szepsenwol et al. 2026). Such developmental histories are highly relevant in the context of childhood exposure to war and terrorism, which can undermine a child's basic sense of safety and protection, fostering persistent perceptions of threat, unpredictability, and danger (Betancourt et al. 2012; Halevi et al. 2016; Joshi and O’Donnell 2003). However, despite the large number of children worldwide who are exposed to war‐related trauma, relatively little is known about the long‐term implications of childhood war‐related insecurity for adult functioning. This gap is particularly salient in contexts of repeated or ongoing exposure to war and terrorism across the life course. For mothers, this issue is especially pertinent, as early experiences of threat may be reactivated when individuals who were exposed to danger as children become caregivers themselves and are once again confronted with external danger, raising the possibility of intergenerational transmission of stress and vulnerability.

Finally, theoretical perspectives on cumulative adversity suggest that the co‐occurrence of distinct trauma types, such as interpersonal dysfunction within the family (ACEs) and childhood experiences of war‐related insecurity, may exert a compounding effect on adult psychopathology (Cloitre et al. 2009; Evans et al. 2013). According to the poly‐victimization framework, an increased burden of diverse traumatic exposures can overwhelm an individual's regulatory capacities more profoundly than a single type of adversity (Finkelhor et al. 2007). Despite this, little is known about the potential combined impact of ACEs and childhood exposure to war‐related threats, or how these distinct yet intersecting forms of adversity jointly relate to the maintenance or escalation of PTSS when individuals face renewed, high‐intensity stressors in adulthood.

Accordingly, in this paper, ACEs and childhood war‐related insecurity are conceptualized as distinct but complementary dimensions of childhood adversity. Whereas ACEs primarily reflect disruptions within the caregiving environment, childhood war‐related insecurity reflects exposure to persistent external threat and compromised safety. Exploring both dimensions allows for the assessment of their unique and combined contributions to maternal PTSS.

## 2 The Present Study

The present study examined war‐related PTSS among mothers of infants during the Israel–Hamas war, which began with the Hamas attack on Israel on October 7, 2023. Maternal PTSS were assessed at two time points during the ongoing war: the first assessment was conducted in March 2024, approximately 5 months after the initial attack, during an intensive phase of the conflict; the second was conducted in May 2025, while the war was still ongoing. Within this context of sustained collective threat, this study focused on how mothers' developmental histories of adversity relate to maternal war‐related PTSS during early motherhood.

Childhood adversity was operationalised using two conceptually distinct dimensions: (a) ACEs and (b) childhood war‐related insecurity. We focused on both the independent contributions of each adversity dimension and their co‐occurrence, using a four‐group typology: No adversity (No ACEs/No childhood war‐related insecurity), ACEs only, Childhood war‐related insecurity only, and Combined (ACEs + Childhood war‐related insecurity). Building on stress sensitisation frameworks, we tested whether the three childhood adversity groups moderated the prospective association between contemporary cumulative war exposure and PTSS at follow‐up (T2), while accounting for baseline PTSS (T1) and sociodemographic covariates. This approach allowed for an examination of whether the link between contemporary war exposure and the severity of PTSS varied as a function of developmental history, with a particular focus on the potential impact of cumulative childhood adversity (i.e., the Combined group).

We hypothesised that:

1. Histories of childhood adversity would be associated with higher PTSS compared to no such histories, with the highest symptom levels expected among mothers reporting both ACEs and childhood war‐related insecurity.
2. Greater contemporary cumulative war exposure would prospectively predict higher PTSS at T2, above and beyond the stability of baseline PTSS.
3. Childhood adversity would moderate the prospective association between contemporary cumulative war exposure and PTSS at T2. Specifically, drawing on the stress sensitisation framework, we expected the association between contemporary cumulative war exposure and PTSS to be more pronounced among mothers with a history of childhood adversity, with the strongest moderation effect anticipated in the Combined (ACEs + Childhood war‐related insecurity) group.

## 3 Method

### 3.1 Participants and Procedure

The participants were recruited as part of a longitudinal study examining maternal and infant well‐being during the Israel–Hamas war. Recruitment was conducted via social media platforms (e.g., Facebook, Instagram, WhatsApp) targeting virtual communities for mothers. The inclusion criteria were: (a) being a mother to an infant aged 1–12 months, (b) age 18 or older, (c) residing in Israel, and (d) proficiency in Hebrew. The exclusion criteria included preterm birth (< 36 weeks), multiple births, and/or diagnosed infant developmental abnormalities. The data were collected at two time points using an online survey questionnaire administered in Hebrew.

The first assessment (T1) took place in March 2024, approximately 5 months after the October 7, 2023, attacks. At the conclusion of T1, participants were asked for consent to be contacted for a follow‐up assessment. The second assessment (T2) was conducted in May 2025, with only the participants who had previously agreed to participate in the longitudinal phase. To maintain anonymity while enabling data integration, at T1, participants were provided with a unique personal identifier code, which they were asked to use when completing the survey at the second assessment. At T1, 792 mothers completed the survey. Of these, 394 also completed the T2 assessment (49.7%). Attrition analyses indicated that the 394 mothers who completed the T2 assessment did not differ from the 398 who did not continue in terms of any of the sociodemographic variables (maternal age, education, income, or parity) or the primary study variables, including T1 initially war exposure, childhood adversity, and childhood war‐related insecurity (all *p*s > 0.05).

### 3.2 Sample Characteristics

The final sample consisted of 394 mothers. Maternal age ranged from 23 to 47 (*M* = 33.69, SD = 4.75). Most participants were married or in a spousal relationship (94.7%), held an academic degree (89.6%), and reported average or above‐average income (89.4%). In terms of parity, 47.5% of the participants were primiparous. Infant age at T1 ranged from 1 to 12 months (*M* = 5.29, SD = 3.07). The second assessment (T2) was conducted approximately 14 months after T1, when the infants were aged between 14 and 26 months (*M* = 19.29, SD = 3.07).

### 3.3 Ethical Considerations

The study was approved by the Institutional Review Board (IRB) (Approval #: 032,404). All the participants provided electronic informed consent before each assessment wave. They were informed that their participation was voluntary, that they could withdraw from the study at any time, and that their responses would remain confidential.

### 3.4 Measures

#### 3.4.1 War‐Related Exposure

War‐related exposure was assessed at both time points using a checklist of war‐related events adapted from Ring et al. (2024). At T1, exposure was measured using 17 items that addressed the October 7, 2023, attack and the subsequent months of war (Chasson et al. 2025; Ring et al. 2024; see Supplementary Appendix S1). These items assessed a broad range of war‐related experiences, including direct and indirect exposure to violent events (e.g., personally witnessing the October 7 attack in Israel or experiencing rocket attacks in one's area of residence), bereavement and loss (e.g., the death or injury of a close family member or friend), disruptions to daily life (e.g., displacement from home), and ongoing security‐related stressors (e.g., a spouse or partner being recruited for military service).

At T2, participants completed a follow‐up version of the same checklist, adapted to capture war‐related exposures that had occurred since the initial assessment. The T2 checklist consisted of 15 items that captured new or ongoing war‐related stressors experienced over the intervening year, including continued exposure to security threats, personal or familial involvement in security forces, physical and psychological injuries, and displacement (see Supplementary Appendix S2). All items were answered using a dichotomous (Yes/No) response format. For each wave, exposure was quantified using a cumulative counting method: a total cumulative score was calculated by summing all affirmative responses, with higher scores indicating a greater number of contemporary war‐related experiences. Specifically, the T1 score reflected initial war exposure. The T2 score reflected contemporary cumulative war exposure, calculated as the sum of exposure reported at T1 and exposure reported at T2, thereby capturing the overall burden of war‐related experiences accumulated across the study period.

#### 3.4.2 Childhood Adversity

Childhood adversity was assessed using the 10‐item *Adverse Childhood Experiences (ACEs) Questionnaire* (Felitti et al. 1998), which measures exposure to various forms of abuse occurring before the age of 18 (physical, emotional, sexual), neglect (physical, emotional), and household dysfunction (e.g., parental divorce, substance abuse, mental illness). The participants responded to each item in a binary (Yes/No) format, and a cumulative ACEs score was calculated by summing the affirmative responses (range: 0–10). The distribution of ACEs scores in the sample is presented in Supporting Information S1: Table S1.

Because, to our knowledge, there is currently no validated measure for specifically evaluating childhood war‐related insecurity, developmental exposure to chronic threat and lack of protection related to war or terrorism before age 18 was assessed using a single retrospective researcher‐developed item: ‘During your childhood (before age 18), did you feel unsafe or unprotected due to direct or indirect exposure to war or terrorism?’ This item was administered in parallel to, but was analytically distinct from, the ACEs Questionnaire.

Based on the responses to the ACEs Questionnaire and this childhood war‐related insecurity item, a typology was created to categorise the longitudinal sample (*N* = 394) into four mutually exclusive groups: (1) No adversity (No ACEs/No Childhood war‐related insecurity; *N* = 185, 47.0%), which represented participants with no reported childhood adversity; (2) ACEs only (*N* = 121, 30.7%), which represented participants with at least one ACE but no childhood war‐related insecurity; (3) Childhood war‐related insecurity only (*N* = 39, 9.9%), which represented participants who reported war‐related insecurity without other ACEs; and (4) Combined (ACEs + Childhood war‐related insecurity; *N* = 49, 12.4%), which represented those with both forms of adversity.

#### 3.4.3 Posttraumatic Stress Symptoms (PTSS)

Maternal PTSS were assessed at both T1 and T2 using the *PTSD Checklist for DSM‐5* (PCL‐5; Weathers et al. 2013). This 20‐item self‐report measure evaluates the frequency of PTSS over the past month. In the present study, the participants were instructed to rate their symptoms in relation to the events of October 7, 2023, in Israel and the ongoing war. The participants rated each symptom on a 5‐point Likert scale ranging from 0 (*never*) to 4 (*very often*). For the primary analyses, a mean (*M*) symptom score was calculated, with higher scores reflecting greater symptom severity. Additionally, the total PCL‐5 sum score was used to assess clinical risk, based on the standard diagnostic cutoff score of ≥ 31 (Bovin et al. 2016). The scale demonstrated high internal consistency with the current sample, with a Cronbach's alpha of 0.91 at T1 and 0.93 at T2.

#### 3.4.4 Sociodemographic Characteristics

The participants provided information regarding maternal and infant age, education level, marital status, parity (primiparous vs. multiparous), and employment status. Socioeconomic status was assessed based on monthly household income relative to the national average. These background variables were assessed at the initial wave and updated as needed during the T2 follow‐up to account for any changes in the participants' status.

### 3.5 Analytic Strategy

#### 3.5.1 Analytic Strategy

The data analyses proceeded in several stages. First, descriptive statistics were computed to characterise maternal PTSS across the four childhood adversity typology groups at T1 and T2. Chi‐square tests were used to compare the proportion of mothers meeting the PCL‐5 diagnostic cutoff for probable PTSD (≥ 31) across the groups at each time point. Preliminary analyses were conducted in SPSS Version 29 and included bivariate correlations between PTSS at T2 and key sociodemographic variables (maternal age, education, economic status, employment status, parity, and child age). The longitudinal analyses were conducted among participants who completed both assessment waves (*N* = 394). Within this longitudinal sample, item‐level missing data were examined prior to the prospective analyses. Little's missing completely at random (MCAR) test was non‐significant, χ^2^ (3) = 5.53, *p* = 0.137, supporting the missing at random (MAR) assumption. Accordingly, full information maximum likelihood (FIML) estimation was used to handle item‐level missingness within the longitudinal sample.

The distributions of the continuous variables were also inspected prior to the prospective analyses. To account for the moderate positive skewness of contemporary cumulative war exposure, the structural equation models were estimated using robust maximum likelihood (MLR), which provides robust standard errors and test statistics in the presence of non‐normality. Prospective analyses were conducted using structural equation modelling implemented with the *lavaan* package (Rosseel 2012), with PTSS at T2 as the outcome, and baseline PTSS (T1) included as a covariate to model symptom change over time. The contemporary cumulative war exposure variable represented the overall burden of war‐related experiences (i.e., across T1 and T2). In the second stage, a main effects model was used to examine the associations among childhood adversity typology (i.e., group), contemporary cumulative war exposure, and PTSS. Childhood adversity typology was modelled as a four‐level categorical variable with mutually exclusive groups, using the No‐adversity group as the reference category. In the third stage, interaction terms between adversity typology and contemporary cumulative war exposure were added to test whether the prospective association between contemporary cumulative war exposure and PTSS varied by childhood adversity. The results are reported using standardized and unstandardised estimates, standard errors, 95% confidence intervals, and *p*‐values. Harman's single‐factor test was used to evaluate the potential influence of common method bias associated with the exclusive use of self‐report measures.

## 4 Results

### 4.1 Preliminary Analyses

Table 1 presents the descriptive statistics and bivariate correlations among the primary study variables. We examined the bivariate correlations between maternal PTSS at T2 and key sociodemographic variables (maternal age, education, economic status, parity, and child age). PTSS were significantly associated with lower economic status (*r* = −0.19, *p* < 0.001) and being multiparous (*r* = 0.11, *p* = 0.028). No significant associations were observed for maternal age, education, or child age (*r*s = −0.07 to 0.02, *p*s > 0.21). Based on these findings, economic status and parity were included as covariates in all subsequent prospective analyses. In addition, Harman's single‐factor test showed that the first unrotated factor accounted for 32% of the total variance, indicating that common method bias was unlikely to account for the observed associations.

**TABLE 1:** Descriptive statistics and bivariate correlations among the primary study variables.

Variable	M	SD	1	2	3	4	5
1. PTSS (T1)	2.264	0.672	—				
2. PTSS (T2)	0.999	0.708	0.651***	—			
3. Contemporary cumulative war exposure	7.025	3.077	0.165**	0.299***	—		
4. Cumulative ACEs score	1.157	1.592	0.196***	0.211***	0.127*	—	
5. Childhood war‐related insecurity	0.223	0.417	0.158**	0.160**	0.085	0.422***	—

*Note:* Childhood war‐related insecurity was coded as 0 = no and 1 = yes. Correlations were calculated using pairwise deletion.

Abbreviations: ACEs = adverse childhood experiences; PTSS = posttraumatic stress symptoms.

^*^ *p* < 0.05.

^**^ *p* < 0.01.

^***^ *p* < 0.001.

### 4.2 Clinical Risk for PTSD

To evaluate the clinical significance of the findings, we examined the proportion of women who met or exceeded the established diagnostic cutoff of 31 on the PCL‐5. This analysis aimed to determine whether childhood adversity typology was associated with an increased likelihood of reaching clinical levels of posttraumatic distress during the Israel–Hamas war. Chi‐square analyses revealed significant group differences in clinical risk at both time points. At T1, approximately 5 months into the war, clinical risk differed significantly by group, χ^2^ (3) = 7.86, *p* = 0.049. The highest prevalence of probable PTSD was observed in the Combined group (ACEs + Childhood war‐related insecurity), where 25 of 49 mothers (51.0%) met the clinical threshold, compared with 43 of 121 (35.5%) in the ACEs only group, 13 of 39 (33.3%) in the Childhood war‐related insecurity only group, and 55 of 185 (29.7%) in the No‐adversity (No ACEs/No childhood war‐related insecurity) group.

By T2, these group differences had become more pronounced, χ^2^ (3) = 15.14, *p* = 0.002. While the overall prevalence of clinical‐level symptoms declined across the sample, the Combined group maintained a strikingly high rate of probable PTSD, with 20 of 49 mothers (41.7%) meeting the clinical threshold. In contrast, clinical rates were substantially lower in three groups: ACEs only (29 of 121, 25.4%), Childhood war‐related insecurity only (8 of 39, 22.2%), and No‐adversity (27 of 185, 15.7%). These results indicate that a combination of childhood interpersonal adversity and childhood war‐related insecurity is associated with a persistent and heightened risk for clinical PTSD under conditions of ongoing threat.

### 4.3 Descriptive Statistics: PTSS by ACEs Typology at T1 and T2

**FIGURE 1:**
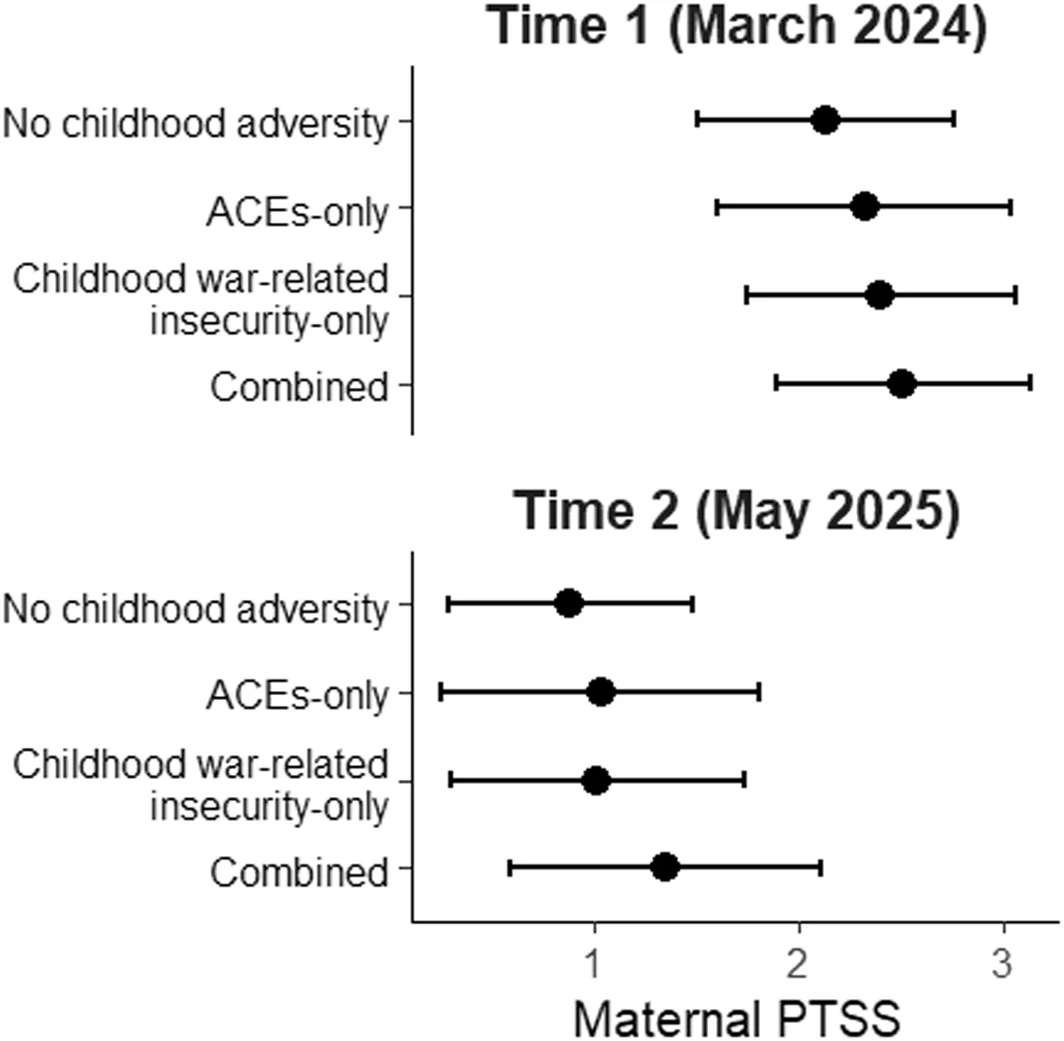
Mean levels of PTSS at T1 and T2 by ACEs typology (*N* = 394). Note. PTSS = posttraumatic stress symptoms; ACEs = adverse childhood experiences; Combined = ACEs + Childhood war‐related insecurity.

The descriptive statistics for PTSD symptoms by ACEs typology (group) at T1 and T2 are presented in Table 2 and illustrated in Figure 1. At T1, mean PTSS differed across groups, with the lowest mean observed in the No adversity (No ACEs/No childhood war‐related insecurity) group (*M* = 2.12, SD = 0.62) and progressively higher means in the ACEs only (*M* = 2.34, SD = 0.71), Childhood war‐related insecurity only (*M* = 2.43, SD = 0.68), and Combined (ACEs + Childhood war‐related insecurity) (*M* = 2.57, SD = 0.66) groups. At T2, PTSD symptom levels declined overall across groups but maintained a similar rank‐order pattern. Mean PTSS were lowest in the No adversity (No ACEs/No childhood war‐related insecurity) group (*M* = 0.88, SD = 0.60) and highest in the Combined (ACEs + Childhood war‐related insecurity) group (*M* = 1.35, SD = 0.76). These descriptive findings suggest both overall symptom reduction over time and persistent group differences associated with cumulative developmental and contextual adversity.

**TABLE 2:** Descriptive statistics of maternal PTSS by ACEs typology at T1 and T2.

ACEs/Childhood war‐related insecurity typology	*N*	*M* T1	SD T1	*M* T2	SD T2
No adversity (No ACEs/No childhood war‐related insecurity) (control group)	185	2.13	0.63	0.88	0.60
ACEs only	121	2.32	0.72	1.03	0.78
Childhood war‐related insecurity only	39	2.40	0.66	1.01	0.72
Combined (ACEs + childhood war‐related insecurity)	49	2.51	0.62	1.35	0.76

*Note:* PTSS declined overall from T1 to T2 but remained highest in the Combined (ACEs + Childhood war‐related insecurity) group at both time points.

### 4.4 PTSS at T2: Prospective Effects of Contemporary Cumulative War Exposure and Childhood Adversity Typology

**FIGURE 2:**
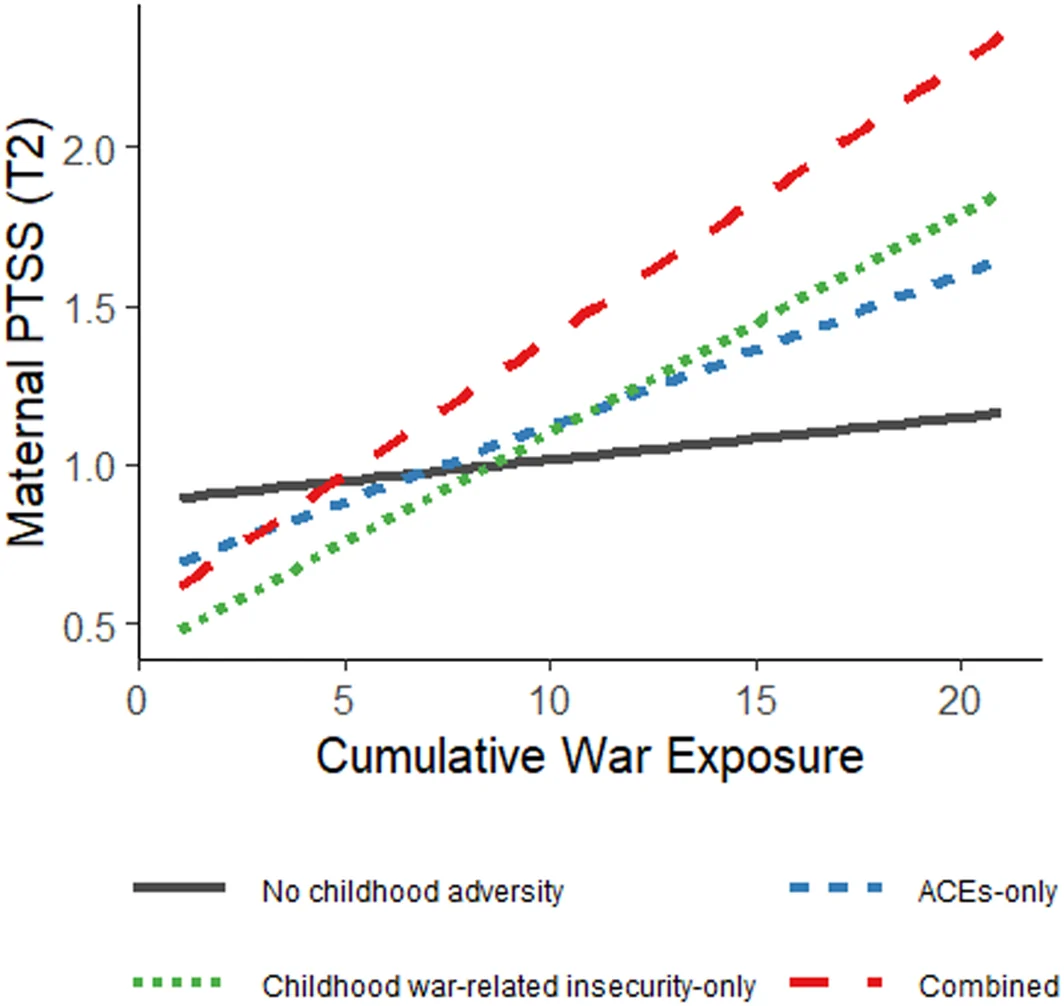
Predicted maternal PTSS at T2 as a function of contemporary cumulative war exposure and childhood adversity typology (*N* = 394). Note. PTSS = posttraumatic stress symptoms; ACEs = adverse childhood experiences; Combined = ACEs + Childhood war‐related insecurity. Results adjusted for baseline PTSS (T1), economic status, and parity.

To examine the prospective predictors of maternal PTSS at T2, we estimated structural equation models using FIML to account for missing data (*N* = 394). We first estimated a model that included only main effects: baseline PTSS at T1, demographic covariates (economic status and parity), childhood adversity typology, and contemporary cumulative war exposure. We then estimated a second model that additionally included the interaction terms between ACEs typology and contemporary cumulative war exposure. The results from the model analysis are presented in Table 3. As expected, maternal PTSS at T1 was the strongest predictor of PTSS at T2 (*β* = 0.59, SE = 0.04, 95% CI [0.54, 0.71], *p* < 0.001), indicating substantial symptom stability over time. Among the demographic covariates, multiparity and lower economic status were associated with higher PTSS (multiparity: *β* = 0.09, SE = 0.05, 95% CI [0.02, 0.23], *p* = 0.017; economic status: *β* = −0.08, SE = 0.04, 95% CI [−0.15, −0.00], *p* = 0.050). Regarding childhood adversity, a significant main effect emerged for the Combined (ACEs + Childhood war‐related insecurity group) (*β* = 0.09, SE = 0.08, 95% CI [0.02, 0.35], *p* = 0.028), indicating higher PTSS relative to the reference groups. In addition, contemporary cumulative war exposure was positively associated with PTSS (*β* = 0.18, SE = 0.01, 95% CI [0.02, 0.06], *p* < 0.001). The model also revealed a significant interaction between the Combined (ACEs + Childhood war‐related insecurity) typology and contemporary cumulative war exposure (*β* = 0.28, SE = 0.03, 95% CI [0.02, 0.13], *p* = 0.006). As illustrated in Figure 2, higher contemporary cumulative war exposure was associated with a steeper increase in maternal PTSS at T2, especially among mothers in the combined adversity group. In contrast, the interaction terms for the ACEs only (*β* = 0.18, *p* = 0.083) and the Childhood war‐related insecurity only (*β* = 0.20, *p* = 0.058) groups reached only trend‐level significance.

**TABLE 3:** Prospective main and interaction effects of childhood adversity typology and contemporary cumulative war exposure on maternal PTSS at T2 (*N* = 394).

	*β*	B	SE	*Z*	95% CI	*p*
Main effects						
PTSS (T1)	0.593	0.625	0.041	15.12	[0.54, 0.71]	< 0.001
Economic status	−0.076	−0.078	0.039	−1.96	[−0.15, −0.00]	0.050
Parity	0.090	0.128	0.053	2.39	[0.02, 0.23]	0.017
ACEs only	0.003	0.005	0.062	0.08	[−0.12, 0.13]	0.935
Childhood war‐related insecurity only	−0.026	−0.061	0.094	−0.65	[−0.25, 0.12]	0.515
Combined (ACEs + childhood war‐related insecurity)	0.087	0.186	0.085	2.19	[0.02, 0.35]	0.028
Contemporary cumulative war exposure (T1+T2)	0.185	0.042	0.009	4.81	[0.02, 0.06]	< 0.001
Interactions						
ACEs only × contemporary cumulative war exposure	0.182	0.034	0.020	1.73	[−0.00, 0.07]	0.083
Childhood war‐related insecurity only × contemporary cumulative war exposure	0.196	0.056	0.029	1.89	[−0.00, 0.11]	0.058
Combined (ACEs + childhood war‐related insecurity) × contemporary cumulative war exposure	0.281	0.073	0.026	2.77	[0.02, 0.13]	0.006

Abbreviations: ACEs = adverse childhood experiences; PTSS = posttraumatic stress symptoms.

Because the primary analyses were based on a categorical childhood adversity typology, we conducted a supplementary sensitivity analysis using a dimensional approach to childhood adversity. Specifically, the continuous ACEs score and childhood war‐related insecurity were entered as separate predictors, together with their interactions with contemporary cumulative war exposure. The overall pattern of findings remained largely consistent: childhood war‐related insecurity, but not using the continuous ACEs score, moderated the association between contemporary cumulative war exposure and PTSS at T2 (Supporting Information S1: Table S2).

## 5 Discussion

The present two‐wave study examined the prospective associations between childhood adversity, contemporary cumulative war exposure, and war‐related PTSS among mothers during the first two postpartum years. Our findings are consistent with the stress sensitisation framework (Hammen et al. 2000; Monroe and Harkness 2005) and indicate that the association between contemporary war exposure and maternal PTSS differs according to the mother's developmental history of adversity. Notably, mothers with a co‐occurrence of ACEs and childhood war‐related insecurity exhibited heightened vulnerability to the impact of the ongoing Israel–Hamas war. This was evidenced by both a significantly higher prevalence of probable PTSD and a stronger prospective link between contemporary cumulative war exposure and symptom severity at follow‐up.

Consistent with our hypotheses, PTSS levels declined over time. Although this pattern could reflect normative adaptation or habituation to the prolonged stress of war (Levin et al. 2025), other methodological and contextual factors may also have contributed to the observed decline. Importantly, despite the reduction in mean PTSS levels, substantial heterogeneity in symptom trajectories remained, with differences observed across the four childhood adversity groups. Mothers in the Combined adversity group (ACEs + Childhood war‐related insecurity) maintained a strikingly high rate of probable PTSD (41.7%), even 14 months after the initial assessment. These findings align with stress sensitisation models, which propose that early adversity primes biological and psychological stress systems towards heightened threat sensitivity (Hammen et al. 2000; McLaughlin et al. 2010; Monroe and Harkness 2005).

This sensitisation may be particularly potent in the context of early motherhood. The first 2 years of motherhood constitute a unique developmental window characterised by a heightened psychological focus on ensuring the infant's safety and well‐being (Gelaye et al. 2016; Miller et al. 2024). During this stage, mothers are inherently more attuned to potential environmental threats as part of their caregiving role. However, for mothers with a history of adversity, this normative vigilance may intersect with a pre‐existing ‘lowered threshold’ for threat. When faced with high‐intensity external stressors such as war, the ongoing demands of caring for a young child, especially those from infancy to toddlerhood age, may exacerbate the reactivation of latent schemas of helplessness and danger (Chasson, Borelli, et al. 2025). For these mothers, the internal drive to provide a secure environment for their child may conflict with a persistent personal sense of insecurity, potentially contributing to the symptomatic stability observed in our data (Pynoos et al. 1999). In addition, the implications of high levels of PTSS symptoms are grave during this period when young children are most reliant on their caregivers for emotion co‐regulation (Callaghan and Tottenham 2016; Feldman 2012), as well as the time period during which attachment relationships are forged (Ainsworth et al. 1978).

A central contribution of this study is the identification of heightened vulnerability associated with the co‐occurrence of distinct forms of childhood adversity. Our results suggest that the accumulation of interpersonal household adversity (ACEs) and external war‐related insecurity in childhood may overwhelm an individual's regulatory capacities more profoundly than a single type of adversity might (Evans et al. 2013; Finkelhor et al. 2007). The moderation analysis in this study revealed that higher contemporary cumulative war exposure was associated with a steeper increase in PTSS, especially for mothers in the Combined childhood adversity group. This finding supports the poly‐victimization framework (Finkelhor et al. 2007), which suggests that a diversity of traumatic exposures, encompassing both proximal household stressors and distal environmental threats, erodes the foundational sense of safety. While ACEs typically represent a breach of the ‘proximal’ safe base (the family), childhood war‐related insecurity represents a breach of the ‘distal’ safe world. When both are compromised, the individual may develop an enduring perception of the world as inherently malevolent and unpredictable (McLaughlin and Lambert 2017).

One possible interpretation of the present findings is that the ongoing Israel–Hamas war may have acted as a contemporary context in which early‐formed beliefs about safety and threat became particularly salient. For mothers with a history of cumulative adversity, the current reality of sirens, loss, and insecurity may have been experienced as a continuation of their earlier experiences of threat rather than solely as a present‐day crisis. This interpretation may help explain why, in the Combined adversity group primarily, ongoing war exposure was prospectively linked to increased PTSS. One plausible possibility is that these individuals' internal working models of safety were more vulnerable to reactivation under conditions of renewed existential threat, leaving them with fewer psychological resources for coping with ongoing adversity.

### 5.1 Clinical Implications

The persistence of clinical‐level PTSS among mothers with cumulative adversity has significant implications for both maternal well‐being and infant development. Maternal PTSS following war‐related trauma has been linked to caregiving‐related alterations, including reduced emotional availability and disruptions in the parent–infant relationship (Chasson et al. 2025; Feldman and Vengrober 2011). The observation that nearly half of the mothers in the Combined adversity group reported symptoms that exceeded the clinical threshold at follow‐up suggests a pattern of persistent, rather than transient, distress. While posttraumatic reactions following acute events often subside over time, the stability of elevated symptoms in this group points to a potentially enduring vulnerability during the early years of motherhood. This underscores the importance of trauma‐informed postpartum care. Screening for childhood adversity, including both household ACEs and childhood war‐related insecurity, may help identify mothers who are at the highest risk for persistent PTSS and who may require more intensive, long‐term psychological support.

Although the present study focused specifically on war‐related PTSS, childhood adversity has also been linked to other forms of maternal psychological distress, including depression and anxiety (Racine et al. 2021). Therefore, comprehensive mental health assessment during the postpartum period may help identify mothers who are experiencing a broader range of psychological difficulties following prolonged war exposure. In the context of ongoing war, such assessments should be accompanied by sustained, trauma‐informed interventions that extend beyond immediate crisis response and, over time, address the evolving mental health needs of mothers. Integrating evidence‐based mental health services into routine maternal and child healthcare may promote earlier identification of vulnerable mothers while supporting both maternal well‐being and the developing parent–child relationship.

### 5.2 Limitations and Future Directions

Despite the longitudinal design, several limitations warrant consideration. First, childhood adversity was assessed retrospectively via self‐report, and therefore, the data may be subject to recall bias. In particular, mothers' current experiences during the ongoing war may have influenced how they recalled or interpreted their childhood experiences, especially those related to childhood war‐related insecurity. Nevertheless, the prospective association with later PTSS suggests that these reports captured clinically meaningful individual differences. Furthermore, although childhood war‐related insecurity was assessed using a single retrospective item developed for this study, allowing us to examine an understudied dimension of adversity, this approach precluded the assessment of reliability and severity. Similarly, although the childhood adversity typology was theory‐driven, categorising adversity may have captured less variability than a dimensional approach.

The interpretation of the findings is further limited by the lack of assessment of several factors that may shape maternal psychological adjustment following war‐related trauma, including postpartum depression, birth‐related PTSD, obstetric characteristics, and detailed information regarding mental health treatment. Likewise, although infant age was included as a covariate, we did not examine infant characteristics or developmental outcomes, which may interact with maternal childhood adversity and war‐related PTSS over time.

Generalisability should also be considered. The sample was characterised by relatively high socioeconomic status, which suggests that the observed associations may differ in more socioeconomically disadvantaged populations. In addition, the prevalence of childhood adversity should be interpreted in light of the specific study population and the ACEs instrument used, as prevalence estimates vary across populations and assessment approaches. Although the participants retained at follow‐up did not differ on the measured sociodemographic characteristics or baseline study variables from those who were lost, selective attrition related to unmeasured factors cannot be ruled out. Finally, because the childhood adversity groups varied in size, with relatively fewer participants in the Childhood war‐related insecurity only and Combined groups, statistical power may have been reduced for the comparisons involving these smaller groups.

To further clarify the pathways to resilience following cumulative developmental and contextual adversity, future research should develop and validate multidimensional measures of childhood war‐related insecurity, compare the categorical and dimensional conceptualizations of childhood adversity, incorporate observational assessments of caregiving alongside biological markers of stress regulation, and include fathers, other caregivers, and protective factors, such as social support and benevolent childhood experiences.

## 6 Conclusion

In conclusion, this study demonstrates that the psychological toll of war on mothers is not distributed equally; rather, it is closely associated with developmental histories of trauma. For mothers with a history of cumulative adversity, the ongoing Israel–Hamas conflict appeared to act as a significant predictor of symptomatic persistence, consistent with the stress sensitisation framework. These findings highlight the need for a holistic approach to addressing maternal mental health in conflict zones, recognising that such crises may be experienced through the lens of earlier developmental experiences. Supporting the mental health of sensitised mothers is a critical step in breaking the potential intergenerational cycle of trauma and ensuring the healthy development of the next generation, especially during times of war.

## Funding

This work was supported by The Foundation for the Contemporary Family Grant to J.L. Borelli and M. Chasson, and by The U.S.‐Israel Binational Science Foundation (BSF) Research Grant to J.L. Borelli and D. Shai.

## Ethics Statement

The study protocol was approved by the Institutional Review Board (IRB) of Bar‐Ilan University. All procedures were conducted in accordance with institutional and international ethical standards.

## Consent

Informed consent was obtained from all participants prior to data collection.

## Conflicts of Interest

The authors declare no conflicts of interest.

## Supporting information


Supporting Information S1

## Data Availability

The data that support the findings of this study are available on request from the corresponding author. The data are not publicly available due to privacy or ethical restrictions.
