# Supplementary material for: War‐Related Posttraumatic Stress Symptoms During the First Two Postpartum Years: The Moderating Role of Childhood Adversity
Source: Stress Health. 2026 Aug 23;42(4):e70216. doi: 10.1002/smi.70216 (PMC13500927; doi:10.1002/smi.70216)
Supplement: Supplementary file 1 [file SMI-42-e70216-s001.docx]

**Supplementary Table S1.** *Distribution of ACEs Scores in the Study Sample (N = 394)*

| **ACE score** | ***N*** | **%** |
| --- | --- | --- |
| 0 | 185 | 47.0 |
| 1 | 100 | 25.4 |
| 2 | 46 | 11.7 |
| 3 | 26 | 6.6 |
| 4 | 18 | 4.6 |
| 5 | 10 | 2.5 |
| 6 | 3 | 0.8 |
| 7 | 4 | 1.0 |
| 8 | 1 | 0.3 |
| 10 | 1 | 0.3 |

**Supplementary Table S2.** *Prospective Main and Interaction Effects of Childhood Adversity (Continuous Measure) and Contemporary Cumulative War Exposure on Maternal PTSS at T2 (N = 394)*

|  | **β** | **B** | **SE** | **Z** | **95% CI** | **p** |
| --- | --- | --- | --- | --- | --- | --- |
| **Main Effects** |  |  |  |  |  |  |
| PTSS (T1) | 0.600 | 0.632 | 0.042 | 15.03 | [0.55, 0.71] | <.001 |
| Economic status | −0.070 | −0.072 | 0.047 | −1.54 | [−0.16, 0.02] | .125 |
| Parity | 0.095 | 0.134 | 0.052 | 2.58 | [0.03, 0.24] | .010 |
| Continuous ACEs score | 0.058 | 0.026 | 0.017 | 1.53 | [−0.01, 0.06] | .125 |
| Childhood war-related insecurity | 0.012 | 0.021 | 0.081 | 0.26 | [−0.14, 0.18] | .795 |
| Contemporary cumulative war exposure (T1 + T2) | 0.164 | 0.038 | 0.008 | 4.88 | [0.02, 0.05] | <.001 |
| **Interactions** |  |  |  |  |  |  |
| Continuous ACEs score × Contemporary cumulative war exposure | 0.040 | 0.005 | 0.004 | 1.25 | [−0.00, 0.01] | .211 |
| Childhood war-related insecurity × Contemporary cumulative war exposure | 0.084 | 0.045 | 0.020 | 2.26 | [0.01, 0.08] | .024 |

*Note*. PTSS = posttraumatic stress symptoms; ACEs = adverse childhood experiences. Continuous predictors were mean-centered prior to computing the interaction terms.

**Supplementary Appendix S1.** *War-Related Exposure Checklist Administered at T1*

|  | **Item** |
| --- | --- |
| **1.** | Personally witnessed the October 7 massacre. |
| **2.** | Physically injured during the October 7 massacre. |
| **3.** | Physically injured by a rocket or mortar attack. |
| **4.** | Stayed in a shelter due to rocket attacks. |
| **5.** | A close family member or friend was killed during the massacre or war. |
| **6.** | A close family member or friend was injured during the massacre or war. |
| **7.** | A close family member or friend went missing during the massacre or war. |
| **8.** | A close family member or friend was taken hostage. |
| **9.** | A close family member or friend was injured by a rocket or mortar attack. |
| **10.** | A close family member or friend witnessed the October 7 massacre and shared their experience. |
| **11.** | Personally knew someone who was killed or injured during the massacre or war. |
| **12.** | Exposed to videos depicting the October 7 terrorist attack. |
| **13.** | Exposed to news reports or articles about the October 7 terrorist attack. |
| **14.** | Home damaged by a rocket or mortar attack. |
| **15.** | Moved out of home because of war-related danger. |
| **16.** | Lost home because of the war. |
| **17.** | Spouse or partner was recruited for military service. |

**Instructions.** The participants were asked to indicate whether they had experienced each of the following events since the October 7, 2023, attack in Israel. All the items were answered using a dichotomous (Yes/No) response format.

**Supplementary Appendix S2.** *War-Related Exposure Checklist Administered at T2*

|  | **Item** |
| --- | --- |
| **1.** | Present in a combat zone or at the site of a security-related incident. |
| **2.** | Physically injured during the war or in a security-related incident. |
| **3.** | Psychologically affected due to exposure to the war or a security-related incident. |
| **4.** | Witnessed others being physically injured during the war or in a security-related incident. |
| **5.** | A close family member or friend was physically injured during the war or in a security-related incident. |
| **6.** | A close family member or friend was psychologically affected by the war or a security-related incident. |
| **7.** | A close family member or friend was killed during the war or in a security-related incident. |
| **8.** | A close family member or friend was, or continues to be, held hostage. |
| **9.** | A rocket, missile, or interception fragment landed near my residence. |
| **10.** | Home damaged by a rocket or mortar attack. |
| **11.** | Stayed in a shelter due to rocket attacks. |
| **12.** | Moved out of home because of war-related danger. |
| **13.** | Household income decreased because of the war. |
| **14.** | Served in the military, police, or emergency services during the follow-up period. |
| **15.** | Spouse or partner served in the military, police, or emergency services during the follow-up period. |

**Instructions.** The participants were asked to indicate whether they had experienced each of the following events since completing the T1 assessment. All the items were answered using a dichotomous (Yes/No) response format.
